# Small Cell Lung Cancer Transformation following Treatment in EGFR-Mutated Non-Small Cell Lung Cancer

**DOI:** 10.3390/jcm11051429

**Published:** 2022-03-05

**Authors:** Isa Mambetsariev, Leonidas Arvanitis, Jeremy Fricke, Rebecca Pharaon, Angel R. Baroz, Michelle Afkhami, Marianna Koczywas, Erminia Massarelli, Ravi Salgia

**Affiliations:** 1 Department of Medical Oncology & Therapeutics Research, City of Hope, Duarte, CA 91010, USA; imambetsariev@coh.org (I.M.); jfricke@coh.org (J.F.); rpharaon@coh.org (R.P.); abaroz@pennstatehealth.psu.edu (A.R.B.); mkoczywas@coh.org (M.K.); emassarelli@coh.org (E.M.); 2 Department of Pathology, City of Hope, Duarte, CA 91010, USA; larvanitis@coh.org (L.A.); mafkhami@coh.org (M.A.)

**Keywords:** EGFR, NSCLC, transformation, SCLC, genomics, precision medicine

## Abstract

EGFR-mutated lung adenocarcinoma patients who received tyrosine kinase inhibitors (TKIs) may initially respond to therapy, but over time, resistance eventually occurs. In a small population (5–10%), these patients can have a histological transformation to SCLC. Nine patients with EGFR-mutated lung adenocarcinoma who transformed to SCLC were evaluated at City of Hope. Patient clinical and pathology data, including multiple next-generation sequencing (NGS) results, clinical therapies, histology, and outcomes, were collected across multiple time points. Descriptive statistics were utilized to visualize and interpret the clinical therapeutic timeline and molecular transformation profiles for these patients. All patients received at least one line of EGFR TKI therapies prior to small cell lung cancer transformation, including erlotinib, afatinib, and osimertinib. Two patients also received chemotherapy prior to transformation (one with immunotherapy). The median months to small cell lung cancer transformation was 16 months, ranging from 4–49 months. The median overall survival (OS) was 29 months from diagnosis, with the minimum of 16 months and maximum of 62 months. The majority of patients had EGFR exon 19 deletion (*n* = 7, 77.8%), and no patients had a change of original oncogenic EGFR mutation over the different time points. Though a TP53 mutation was detected in eight patients (88.9%) either at the first biopsy or the subsequent biopsies, an RB1 alteration was only detected in one patient at presentation, and three patients upon subsequent biopsies (*n* = 4, 44.4%). Each patient had a unique molecular profile in the subsequent molecular testing post-transformation, but BRAF alterations occurred frequently, including BRAF rearrangement (*n* = 1), fusion (*n* = 1), and amplification (*n* = 1). Our results showed that EGFR-mutated lung adenocarcinoma to SCLC transformation patients have a unique histological, molecular, and clinical profile over multiple time points, with further heterogeneity that is not currently reported in the literature, and we suggest more work is required to better understand the molecular heterogeneity and clinical outcomes over time for this EGFR TKI resistance subtype.

## 1. Introduction

EGFR-directed tyrosine kinase inhibitors (TKIs) have become the standard of care for EGFR-mutated (EGFRm) patients, with improved outcomes, but most patients eventually progress due to secondary resistance mutations [1,2]. However, in a small percentage of the EGFR-treated population (3–15%), the patient’s tumor has a histological transformation from non-small cell lung cancer (NSCLC), adenocarcinoma in particular, to neuroendocrine differentiated histology, specifically small cell lung cancer (SCLC) [3,4]. Repeat biopsies are now often performed regularly on patients who are undergoing EGFR-TKI therapy to not only detect resistance EGFR mutations, but to also detect any histological transformation that may occur [5]. The clinical history and unique genomic profiles of patients with small-cell lung cancer transformation are still poorly understood—and several studies showed differential findings regarding acquired mutations and treatment responses for this patient population [6,7]. However, early detection and confirmation of this resistance are important because SCLC-chemotherapeutic treatment has been shown to be clinically effective, and may improve outcomes for these patients [3,8]. 

Therefore, our study aimed to detail and catalog the longitudinal sequencing profiles of nine EGFR-mutated patients who had pathology-confirmed small cell lung cancer transformation, as well as identify any biomarker patterns associated with improved survival. 

## 2. Materials and Methods

### 2.1. Patients

A total of nine patients (*n* = 9) were selected who had an initial diagnosis of EGFR-mutated NSCLC, and upon treatment, transformed to histology-confirmed SCLC between 2014 and 2021. Deidentified patient data were obtained with approval by the City of Hope Institutional Review Board under IRB #21458, and in accordance with the Declaration of Helsinki. The data collected from the electronic medical record included patient demographics, stage, age at diagnosis, race, smoking history, date of diagnosis, therapies received, initial histology, transformation histology, dates of diagnosis and transformation, and outcomes. The molecular genomic results were also collected through retrospective chart review based on next-generation sequencing (NGS) tests that were obtained by their primary oncologist during the course of their care at multiple time points. 

### 2.2. Immunohistochemistry and Genomics

Histologic marker data were collected through retrospective data collection from the electronic medical record. Next-generation sequence molecular marker data were collected through retrospective chart review of clinical genomic testing performed as requested by the primary oncology. Liquid and tissue biopsy molecular testing was performed through commercially available platforms, including City of Hope HopeSeq, Foundation Medicine, Guardant Health, Clarient Diagnostic, QUEST Diagnostic, and Mayo Clinic Laboratories. Formalin-fixed paraffin-embedded (FFPE) sections from lung cancer specimens were cut at 4-micron thickness, and deparaffinized in Ventana EZ prep solution/Leica Dewax at 72 C for 16 min. The slides were then subjected to antigen retrieval in Ventana cell conditioning (CC1) solution/Leica ER1ER2 epitope retriever at 96 C for 36 min. Following this, the sections were blocked for non-specific binding of antibodies by incubating with goat Ig block at room temperature for 8 min. This was followed by incubation of the primary antibodies, with dilutions, vendors, and clones as reported in Table 1, at room temperature for 32 min.

After rinsing, the autostainer performs Ventana Ultraview universal and optiview/Leica BOND polymer detection kit DAB detection procedure, which includes successive incubation with DAB inhibitor (3% H_2_O_2_) horseradish peroxidase-linked anti-mouse multimer, DAB chromogen, and substrate and copper enhancer. The procedure results in brown precipitation. This is followed by counterstain with one drop of hematoxylin for 8 min, and one drop of bluing reagent for 4 min. The slides are removed from the autostainer, washed in water with dishwashing detergent, and mounted as per IHC standard operating protocols. All glass slides were reviewed and interpreted by a board-certified pathologist.

## 3. Results

### 3.1. Patients

A total of nine patients diagnosed with NSCLC, and who had an oncogenic EGFR alteration detected through NGS, were seen for follow-up from 2014 through 2021 at City of Hope (Table 2). The median age at metastatic diagnosis was 60 years old, ranging from 35–72 years. More than half of patients were female (*n* = 5, 55.6%), and were Caucasian (*n* = 5, 55.6%) or Asian (*n* = 4, 44.4%), with the majority being never-smokers (*n* = 5, 55.6%). At the time of diagnosis, all nine patients had a histologically-confirmed diagnosis of lung adenocarcinoma and an EGFR mutation. The majority of patients had an EGFR exon 19 deletion (*n* = 7, 77.8%), exon 21 L858R mutation (11.1%), or L861Q (*n* = 1, 11.1%). All patients received at least one line of EGFR TKI therapies prior to small cell lung cancer transformation, including erlotinib, afatinib, and osimertinib. Two patients also received chemotherapy prior to transformation (one with immunotherapy). The median months to small cell lung cancer transformation was 16 months, ranging from 4–49 months. Following transformation, all patients received chemotherapy treatment, with only one patient receiving additional immunotherapy. Three patients enrolled in a clinical trial after failing chemotherapy. EGFR TKI therapy was continued alongside chemotherapy for four patients immediately after transformation, and the remaining had EGFR TKI therapy following the completion of their chemotherapy regimen.

### 3.2. Histological and Genomic Findings

**Figure 1:**
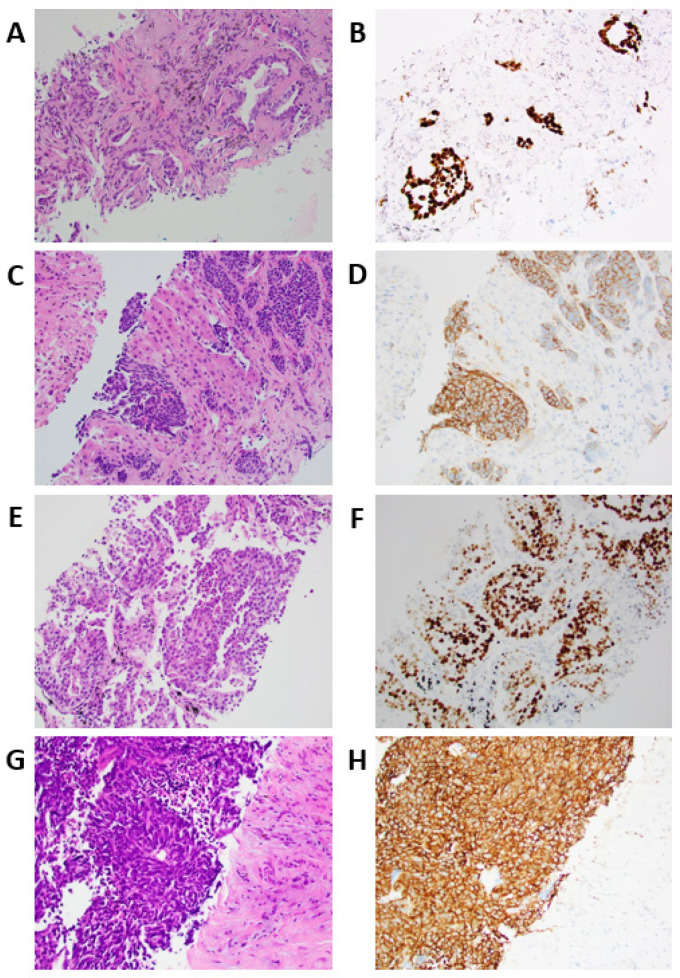
Tissue staining of two patients with transformation. (**A**) H&E stained section of acinar-type adenocarcinoma grade 2, initial diagnosis, H&E ×200 (Patient 9). (**B**) Acinar-type adenocarcinoma, TTF1 stain ×200. (**C**) H&E section, small cell transformation to liver ×200. (**D**) Small cell transformation to liver, synaptophysin stain, ×200. (**E**) H&E stained section of solid-type adenocarcinoma grade 3, initial diagnosis, H&E ×200 (Patient 7). (**F**) Solid-type adenocarcinoma, TTF1 stain ×200. (**G**) H&E section, small cell transformation to liver ×200. (**H**) Small cell transformation to liver, synaptophysin stain ×200.

All nine patients had adenocarcinoma histology upon diagnosis. All eight initially diagnosed adenocarcinomas that were tested for TTF-1 were positive for TTF-1 (*n* = 8, 100%), and in one adenocarcinoma patient, TTF-1 was not performed (Table 3). The site of SCLC transformation biopsy was metastatic in 7/9 cases (77.78%), with the liver being the most common metastatic site (3/9, 33.33%). The median months to transformation was 16 months, with the shortest transformation occurring 4 months from diagnosis, and the longest occurring 49 months after diagnosis. The median overall survival (OS) was 29 months from diagnosis, with the minimum of 16 months, and maximum of 62 months. Upon transformation, all of the patients had histologically-confirmed SCLC (*n* = 9, 100%), and all patients were synaptophysin positive, whereas only six (66.67%) were chromogranin positive (Figure 1).

**Figure 2:**
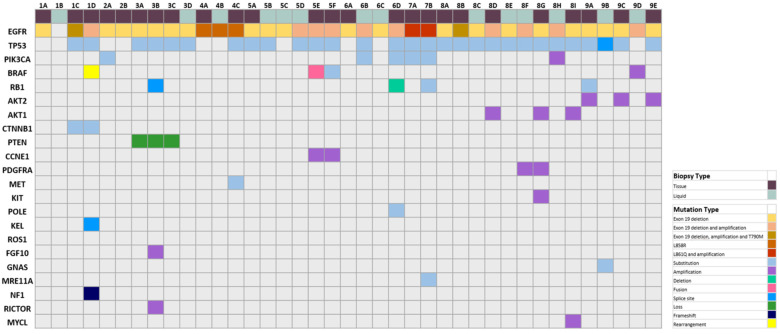
Mutational profile of EGFR NSCLC to neuroendocrine-transformed patients with multiple time points. NGS mutation results from both tissue and liquid biopsy were classified according to mutation subtypes, including EGFR exon 19 deletion; exon 19 deletion and amplification; exon 19 deletion, amplification, and T790M; L858R; L861Q and amplification; and other mutational subtypes, including substitution; amplification; deletion; fusion; splice site; loss; frameshift; rearrangement. The time points of the different molecular testing results are denoted by letters A-I for each patient in sequential order that they were performed.

The majority of patients had EGFR exon 19 deletion (*n* = 7, 77.8%), and no patients had a change of original oncogenic EGFR mutation over the different time points (Figure 2). A TP53 mutation was detected in eight patients (88.9%) either at the first biopsy or at the subsequent biopsies. PIK3CA was the third-most common alteration (*n* = 4, 44.4%), and, interestingly, BRAF alterations occurred frequently in subsequent biopsies, including BRAF rearrangement (*n* = 1), fusion (*n* = 1), and amplification (*n* = 1). RB1 alteration was detected in one patient at presentation, and three patients upon subsequent biopsies (*n* = 4, 44.4%). Patient 1 did not have an EGFR detected by liquid biopsy at timepoint 1B, but upon tissue biopsy analysis at time point 1C, the exon 19 deletion, amplification, and T790M were all detected. The T790M mutation was not detected at 1D, the time point of SCLC transformation following osimertinib treatment, but BRAF rearrangement CTNNB1 substitution, KEL splice site mutation, and NF1 frameshift mutations were found. Patient 3, who had the shortest time to transformation, and longest overall survival, had a PTEN loss at all time points. As well, RB1 splice site 2663 + 1G > A mutation, FGF10 amplification, and RICTOR amplification were detected at the second time point 3B, but RB1 splice site 2663 + 1G > A was no longer detected at time point 3C. 

Patient 5 had the longest time to transformation, with a BRAF fusion prior to transformation, but a BRAF substitution after transformation, and a CCNE1 amplification was detected at both biopsies 5E and 5F. Patients 4 and 7 both had a shorter than median overall survival time, but exhibited different mutational profiles, with patient 4 only exhibiting TP53 and MET substitution, whereas patient 7 had alterations in TP53, PIK3CA, RB1, POLE, and MRE11A. Patient 8 had the most multiple biopsies (nine time points), with EGFR and TP53 mutations detected in all nine. PIK3CA amplification was only detected at 8H, but was not detected in 8I, suggesting a discordance between the liquid and tissue biopsies. AKT1 amplification first occurred in 8D, but was not detected in liquid biopsies until tissue biopsy 8G after the patient received carboplatin/etoposide, osimertinib, and a clinical trial. PDGFRA amplification, however, was detected in liquid biopsy 8F, and confirmed in tissue biopsy 8G. Biopsy 8G was also able to detect a KIT amplification. Patient 9 had the most alteration changes during the different time points. There was a loss of RB1 substitution after time point 9A; TP53 switching from substitution to splice site mutation between time points 9B and 9C; GNAS mutation detected only at time point 9B; and a loss of a TP53 mutation, and detection of BRAF amplification at time point 9D. All nine patients had an acquired mutation that was not present at the time of their first biopsy.

### 3.3. Patient Timelines and Therapies

**Figure 3:**
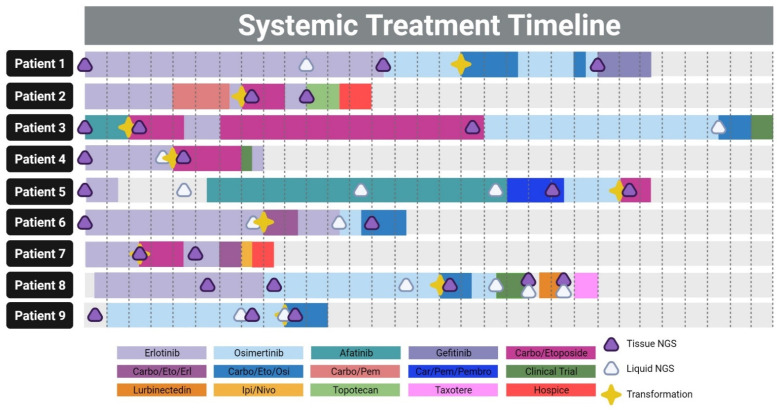
Systemic treatment timeline, the incidence of transformation, and next-generation sequencing time points. The complete history of lines of therapies given for each patient, including the time to transformation, and next-generational sequencing time points, with each box delineating two months. Created with BioRender.com.

The patients’ median overall survival was 29 months, with the longest survival of 62 months, and shortest of 16 months (Figure 3). Patient 1 was on therapy for 51 months, and received erlotinib in the first line, followed by osimertinib, and developed SCLC transformation 34 months after initiating an EGFR TKI. Osimertinib treatment was continued due to a good initial response, and carboplatin/etoposide chemotherapy was initiated alongside targeted therapy for 5 months. However, the patient eventually progressed, and was switched to gefitinib. Patient 2 was initiated on erlotinib, but after 8 months, progressed, and was initiated on concurrent carboplatin/pemetrexed chemotherapy. SCLC transformation was detected 6 months later, after 5 months of chemotherapy and an additional month of erlotinib. The patient was switched to carboplatin/etoposide, and tolerated therapy for 4 months. Erlotinib was attempted to be reinitiated, but the patient quickly progressed and was switched to topotecan, which was also unsuccessful. Patient 2 was put on hospice 9 months after the initial transformation. Patient 3 had the longest survival of 62 months, and was initiated with afatinib therapy, but SCLC transformation was detected after only 4 months, the shortest for our cohort. The patient was treated aggressively with carboplatin/etoposide, and eventually switched back to osimertinib for long-term follow-up. Upon progression, the carboplatin/etoposide/osimertinib regimen was given, and the patient became eligible for a clinical trial.

Patient 4 received erlotinib in the first line with transformation after 8 months, followed by carboplatin/etoposide therapy with a clinical trial where the patient was removed after 1 month. Patient 5 received erlotinib therapy, and was monitored for a period of 8 months with no therapy, but was eventually switched to afatinib, followed by carboplatin/pemetrexed/pembrolizumab and osimertinib. SCLC transformation occurred while on osimertinib therapy after 48 months since initiating an EGFR TKI, the longest in our cohort. Patient 6 received erlotinib, but progressed with transformation, at which time, erlotinib was given in combination with chemotherapy. Osimertinib was administered and combined with carboplatin/etoposide after 2 months. Patient 7 transformed after only 5 months on erlotinib therapy, and was given carboplatin/etoposide. A carboplatin/etoposide/erlotinib combination was attempted, but unfortunately, progression occurred, and the patient was given compassionate care ipilimumab/nivolumab therapy prior to hospice. Patient 8 was initiated on erlotinib, followed by osimertinib, with progression and transformation occurring 16 months into osimertinib therapy. Several lines of therapy were attempted after carboplatin/etoposide/osimertinib, including a clinical trial, lurbinectedin, and taxotere. Patient 9 was initiated on osimertinib for 16 months until transformation, which was subsequently followed by carboplatin/etoposide/osimertinib therapy for 4 months.

## 4. Discussion

EGFR-mutated NSCLC to SCLC transformation has been reported to occur in 3–15% of all EGFR-mutated patients, which suggests an overall incidence of 1% to 4.5% of all NSCLC cases, which underscores the rarity of this phenomenon that has been linked with resistance to EGFR TKI therapy [6,7]. The exact cause and mechanism behind this transformation and resistance to EGFR TKI therapy is not yet completely understood, and though some preclinical models have proposed epithelial-to-mesenchymal transformation (EMT) as a possible mechanism, it is inconclusive that EMT is the driver of resistance [9,10]. Mutations that affect TP53, RB1, PIK3CA, as well as acquired EGFR mutations, such as C797S, have also been reported to be associated with SCLC transformation, but, similar to our cohort, the results are not yet conclusive to arrive at a definitive resistance profile suggesting a possible non-genetic mechanism such as EMT may be at play [6,7,11]. It has also been reported that patients with a triple positive mutational profile of EGFR, TP53, and RB1 had a six-times higher risk of SCLC transformation than those without TP53 and RB1, but, as noted by our cohort and other published results, this is not a requirement to undergo SCLC transformation, suggesting that other pathways, including MAPK, MET, NOTCH-1, and IGFR1, may be involved [10,12]. 

We have shown that EGFR to SCLC transformation may occur at any point during the course of the disease, with a range between 4 months to 49 months—however, the median time to transformation was 16 months, which coincides with the available literature reporting an average time of 17.8 months [3]. Treatment responses following transformation were closely identical to those experienced by de-novo SCLC patients, with an initial response to carboplatin-etoposide therapy, but eventual progression [3,13,14]. Therapeutic options in the SCLC setting showed that few therapies outside of the carboplatin-etoposide regimen were effective. The reason for this may be two-fold: first, more recent results showed that SCLC patients did not receive a long-term overall survival benefit from individual PD-1/PD-L1 therapies, and FDA-approved indications for these drugs were withdrawn [15,16,17,18,19]; secondly, the presence of the founder EGFR mutation in subsequent biopsies of these patients suggests that these tumors retain NSCLC-mutant characteristics, which now poorly respond to immune checkpoint inhibitors [20,21,22]. Approval of atezolizumab alongside carboplatin and etoposide suggests that immunotherapy combination therapies alongside TKI or chemotherapy may be the more tempered approach for EGFR-mutant transformed populations [23]. At the same time, a newer generation of immune checkpoint inhibitors, such as sintilimab, are currently under evaluation, and may offer new avenues of combination therapy [24]. 

EGFR mutant NSCLC patients who undergo SCLC transformation have a poor survival prognosis after transformation. A previous study of 39 patients reported an average survival of 6 months after SCLC transformation [13]. The study also found that smoking status was significantly associated with poorer survival. After SCLC transformation, patients discontinued EGFR TKI treatment, and were treated with systemic chemotherapy due to an increased chemosensitivity of SCLC tumors. Another analysis of 67 patients reported a median overall survival of 10.9 months after SCLC transformation [3]. Patients who were subsequently treated with chemotherapy demonstrated a tumor response, whereas patients treated with immunotherapy (*n* = 17) showed no radiographic response. Ferrer et al. examined EGFR and non-EGFR mutant NSCLC patients who experienced histologic transformation to SCLC. Though non-EGFR patients have been reported to have NSCLC to SCLC transformation, this phenomenon occurs at a much lower frequency, and non-EGFR transformations most often occur in heavy smokers, whereas EGFR-driven SCLC transformation often occurs in former or never-smokers, as reported by Ferrer et al. [25]. They also found that transformation occurred more rapidly in EGFR mutant tumors [25]. Before the transformation, the median overall survival was 28 months in the EGFR mutant group, whereas the median overall survival after transformation was much lower (10 months). This is consistent with the study that reported a median overall survival after SCLC transformation of 10.9 months, but an overall survival since diagnosis of 31.5 months [3]. In comparison, EGFR-mutated NSCLC patients who did not have SCLC transformation have been recently shown to have a median overall survival of 38.6 months [26]. Overall, poor survival is correlated with SCLC transformation, and platinum or taxane chemotherapy treatment often yielded tumor responses [3].

SCLC tumor cell lines have, almost universally, a genomic profile consisting of the inactivation mutation or loss of both tumor suppressor genes TP53 and RB1 [27]. SCLC has a strong association with a history of smoking; however, new research suggests that SCLC never-smoker cells represent a separate biological identity with significantly longer median OS; a lower mutation frequency of RB1; and higher rates of EGFR, MET, and SMAD4 mutations [28]. RB1 inactivation was believed to be responsible for the transformation of EGFRm lung adenocarcinoma to SCLC following EGFR TKIs, similar to de-novo SCLC [29]. Yet, only 44.4% of patients (*n* = 4) in our cohort had an RB1 mutation, whereas 88.9% of patients (*n* = 8) had a TP53 mutation, and 44.4% (*n* = 4) had a PIK3CA alteration. Our dataset matches similar EGFRm lung adenocarcinoma to SCLC transformation studies. One multicenter retrospective study out of China identified TP53 (17/25, 68.0%) as the most prevalent alteration, followed by RB1 (9/25, 36.0%), and PIK3CA (3/25, 12.0%) [30]. Surprisingly, 28% of patients in that study (*n* = 7) did not have a mutation in either TP53 or RB1 at the transformation. There was one patient in our cohort that matched that genotype. A recent study extensively analyzed the characterization of neuroendocrine transformation by evaluating mixed lung adenocarcinoma and SCLC histology, pre-transformation lung adenocarcinomas, post-transformed SCLCs, never-transformed lung adenocarcinomas, and de-novo SCLC. Almost all transformed specimens had a loss in TP53 (93%), with RB1 alterations observed less frequently (63%) [31]. One research conclusion in their study was that neuroendocrine transformation was not directed by mutational events, but rather through lineage plasticity by transcriptional reprogramming. 

Moreover, patients with EGFR/TP53/RB1-mutant lung cancers at diagnosis are at an increased risk of developing SCLC transformation, discontinuing EGFR-TKIs sooner, and a decreased OS [12]. Patient 9, the only EGFR/TP53/RB1 mutated in our cohort, transformed after 16 months of osimertinib, and had a median OS of 22 months. The time to transformation was identical to the median in our cohort, whereas the median OS was less (by 7 months). Early detection of EGFR resistance mechanisms and responses to therapy utilizing non-invasive technology, such as liquid biopsies detecting circulating tumor DNA, may be one tool used in the clinic to monitor these patients [32,33]. Although neuroendocrine transformation has been widely observed in EGFRm lung adenocarcinomas, it is not unique, and has been observed in patients with driver mutations in ALK and ROS1 [4,34]. Additionally, two patients with metastatic lung squamous cell carcinoma were given immunotherapy as second-line therapy following chemotherapy, and later transformed to SCLC [35]. These cases suggest that SCLC transformation is more broadly a resistance mechanism in lung cancer, and necessitates further research.

Our study was limited by the small cohort of patients due to the rarity of the SCLC transformation in the EGFR population. The analysis also only included patients from a single institution, and, in the future, it will be important to collaborate on a larger cohort with different institutions. The availability of genomic results was also limited by the standard of care practice, and future EGFR clinical trials that perform multiple pre- and post-biopsies would be required to further identify the cause of SCLC transformation in this population. The absence of germline mutation analysis was also a limiting factor, and future studies may be needed to further elucidate the relationship between EGFR to SCLC transformation and germline mutations. Nevertheless, our study offers a comprehensive histological, genomic, and clinical assessment of several EGFR to SCLC transformed patients, which will benefit further study of this rare phenomenon. 

## 5. Conclusions

In summary, the histological, clinical, and genomic characteristics of EGFR-mutated patients who transform to SCLC on EGFR therapy are unique and complex, with each individual having a distinct time to transformation without a concrete genomic profile to identify patients at risk for transformation. Though some patients had TP53 or RB1 mutations before transformation, this biomarker was not uniform in our cohort, and this is similar to what is noted in the literature [3,11,36,37]. Furthermore, the clinical outcomes for these patients, though initially showing response to carboplatin and etoposide chemotherapy, eventually end with progression and resistance. However, the one patient who was able to re-initiate EGFR TKI therapy following the chemotherapy regimen had the longest overall survival, which suggests that EGFR TKI therapy may have a role in the post-transformation therapy regimen. The use of immune checkpoint inhibitor therapy showed a poor response following transformation, and is conclusive with the poor efficacy of immune checkpoint inhibitors in EGFR-mutant populations [38,39]. 

In terms of genomics, the founder EGFR mutation was detected in all subsequent tissue biopsies, but was missing in one liquid biopsy. Though TP53, RB1, and PIK3CA are common mutations in SCLC, the presence of these alterations was not uniform in our post-transformation genomic analyses. Therefore, it is important to consider other potential biomarkers that could be indicative of the EGFR to SCLC transformation, such as germline mutational analysis, with germline mutations in lung cancer becoming more commonly detected [40]. Any future additional therapeutic studies for EGFR TKIs should take into consideration the possibility of SCLC transformation, and perform sub-sequential histological examinations where SCLC genomic markers may not be necessarily detected by liquid biopsy.

## Figures and Tables

**Table 1:** Primary antibodies.

Antibody	Vendor	Clone	Dilution
AE1/AE3	Leica	AE1/AE3	Pre-diluted
Chromogranin	Ventana	LK2H10	Pre-diluted
CDX-2	Ventana	ERP2764Y	Pre-diluted
CK7	Cell Marque	OV-TL12/30	Pre-diluted
CK20	Ventana	RAB-MONO	Pre-diluted
CK5/6	Ventana	DS-16B4	Pre-diluted
INSM1	Santa Cruz	A-8	Pre-diluted
p53	Ventana	BP53-11	Pre-diluted
p63	Ventana	4A4	Pre-diluted
p40	Ventana	BC-28	Pre-diluted
Synaptophysin	Ventana	SP11	Pre-diluted
Cam5.2	Ventana	Cam5.2	Pre-diluted
CD56	Leica	CD564	Pre-diluted
TTF-1	Ventana	SP-141	Pre-diluted

**Table 2:** Patient demographics and therapies.

Demographic	Total
Median age at diagnosis (range)	60 (35–72)
Median months to transformation (range)	16 (4–49)
Sex no. (%)	
Male	4 (44.4%)
Female	5 (55.6%)
Race no. (%)	
Caucasian	5 (55.6%)
Asian	4 (44.4%)
Smoking status	
Smoker	4 (44.4%)
Never-smoker	5 (55.6%)
Histology at diagnosis	
Adenocarcinoma	9 (100%)
Initial EGFR mutation	
Exon 19 deletion	7 (77.8%)
Exon 21 (L858R)	1 (11.1%)
L861Q	1 (11.1%)
Therapies received prior to transformation	
Erlotinib	7
Osimertinib	4
Afatinib	2
Carboplatin/pemetrexed	1
Carboplatin/pemetrexed/pembrolizumab	1
Therapies received after transformation	
Carboplatin/etoposide	5
Carboplatin/etoposide/osimertinib	4
Erlotinib	4
Osimertinib	3
Clinical Trial	3
Carboplatin/etoposide/erlotinib	1
Gefitinib	1
Ipilimumab/nivolumab	1
Lurbinectedin	1
Taxotere	1
Topotecan	1

**Table 3:** Histological immunostains at diagnosis and upon transformation.

	Histology at Diagnosis	Histologic Grade at Diagnosis	Histologic Subtype at Diagnosis	Positive Immunostains at Diagnosis	Negative Immunostains at Diagnosis	Months to Transformation	Histology at Transformation	Positive Immunostatins at Transformation	Negative Immunostains at Transformation	Site of transformation Biopsy
1	Adenocarcinoma	2	Acinar	Keratin 7, Napsin, TTF1	Chromogranin, CK20, Synaptophysin	35	small cell carcinoma	synaptophysin, chromogranin, AE1/AE1, Keratin (Oscar),	CK7, CK20, P63, P40, TTF1, Napsin A, CDX2	Left lung
2	Adenocarcinoma	3	Solid	Keratin 7, TTF1	CK20,	16	small cell carcinoma	AE1/AE3, CK7, TTF1, Synaptophysin	Chromogranin, P40	Left back mass (metastatic)
3	Adenocarcinoma	3	Solid	TTF1, CK7	CK20, CDX2	4	small cell carcinoma	CK7, CAM5.2, AE1/AE3, TTF1, Synaptophysin, chromogranin, CD56	CK20	Lymph node, right anterior pericardic (metastatic)
4	Adenocarcinoma	2	Acinar	TTF1		9	small cell carcinoma	MCK, CK7, Synaptophysin, TTF1	Chromogranin	Left retroperitoneal node (metastatic)
5	Adenocarcinoma	2	Acinar	TTF1, Napsin A, CK7, CK20		49	small cell carcinoma	synaptophysin, chromogranin, TTF1, INSM1, p53, CK7	p40, CK5/6, Napsin A, CK20	Liver (metastatic)
6	Adenocarcinoma	N/A	N/A	CK7, TTF1	CK20	16	small cell carcinoma	AE1/AE3, synaptophysin, chromogranin, TTF1		Right lung
7	Adenocarcinoma	2	Acinar	N/A	N/A	6	small cell carcinoma	Keratin 7, keratin 20, CDX2, TTF1, synaptophysin, chromogranin	Napsin A, P40	Liver (metastatic)
8	Adenocarcinoma	3	Solid	TTF1	P63	32	small cell carcinoma	CK7, Synaptophysin, INSM1, TTF1, CDX2, P53	Napsin- A, P40	Supraclavicular, right internal mammary, pericardial phrenic lymph node (metastatic)
9	Adenocarcinoma	2	Acinar	TTF1, CK7, Napsin A	P40	18	small cell carcinoma	CK7, Synaptophysin, chromogranin, CD56, INSM1, TTF1, NapsinA, PanCK	CK20, p63	Liver (metastatic)

## Data Availability

All of the pertinent data for this study were provided within the manuscript.
